# Association Between Vitamin D and Adrenal Parameters with Metabolic and Inflammatory Markers in Polycystic Ovary Syndrome

**DOI:** 10.1038/s41598-019-40653-z

**Published:** 2019-03-08

**Authors:** P. Maidana, A. Fritzler, Y. Mocarbel, M. B. Perez Lana, D. González, M. Rosales, F. González de Chazal, K. Sternberg, E. Lamas Majek, S. Mallea-Gil, E. Genovesi, M. Pelayo, B. Fabre, V. Mesch

**Affiliations:** 1 Universidad de Buenos Aires, Facultad de Farmacia y Bioquímica, Departamento de Bioquímica Clínica - Instituto de Fisiopatología y Bioquímica Clínica (INFIBIOC), Buenos Aires, Argentina; 2 Universidad de Buenos Aires, Hospital de Clínicas “José de San Martín”, División Endocrinología, Buenos Aires, Argentina; 3 Universidad de Buenos Aires, Hospital de Clínicas “José de San Martín”, División Ginecología, Buenos Aires, Argentina; 4 Servicio de Endocrinología, Hospital Militar Central, Buenos Aires, Argentina

## Abstract

Vitamin D deficiency has been related with metabolic alterations in polycystic ovary syndrome (PCOS). As well, hyperactivation of adrenal axis can be programmed early in life and could be related later with PCOS development. Our aim was to establish the relationship between vitamin D and adrenal parameters with metabolic alterations and inflammation markers in PCOS. In 73 patients and 33 controls, 25-hydroxyvitamin D (25-OH-D), total and bioavailable testosterone (TT and bioT), androstenedione (A4), SHBG, cortisol, insulin, and C-reactive protein (hs-CRP) were determined; HOMA and lipid accumulation product (LAP) index were calculated. All parameters were higher in patients than in controls, except for SHBG and 25-OH-D which were lower. Binary regression analysis showed that differences in TT, bioT, A4, insulin and HOMA were independent of body mass index and waist circumference but SHBG, hs-CRP, LAP and 25-OH-D were related to body weight and fat distribution. Binary logistic regression analysis showed that cortisol and 25-OH-D could be associated to PCOS development. Correlations found between LAP and insulin, HOMA and hs-CRP confirm it is a good indicator of metabolic complications. Vitamin D and cortisol association to PCOS development justifies future research to understand the role of vitamin D in PCOS and analyze patient’s perinatal history and its possible relationship with hyperactivation of adrenal axis in adult life.

## Introduction

Hyperandrogenism is a common endocrine disorder in women of reproductive age. Polycystic ovary syndrome (PCOS) is the most frequent etiology with an incidence between 5 and 10% in adolescents and adult women. Given its complex pathophysiology and heterogeneous etiology, the development of PCOS could involve a variety of genetic and environmental factors^1^. Menstrual disorders and infertility are common features in these patients, as well as dermatological alterations^2^. Metabolic disorders include glucose intolerance, insulin resistance^3^ and an atherogenic lipoprotein profile^4^. In turn, insulin resistance, hyperinsulinemia, and dyslipemia are correlated with high abdominal fat deposition^5^. PCOS is also related to functional alterations in adipose tissue: androgen excess can cause adipocytes hypertrophy and both adipose tissue hypertrophy and hyperandrogenism are related to insulin resistance^6^. Considering this, it could be useful to find clinical markers of visceral fat for the screening of metabolic risk in PCOS women^7,8^. In this regard, the use of lipid accumulation product (LAP) index, as an indicator of high risk of developing cardiovascular disease, diabetes and other insulin resistance comorbidities, has been proposed^9,10^. The LAP index is an ordinal scale that combines waist circumference (WC) and triglycerides.

Visceral fat is also considered a condition of low-grade inflammation, with high levels of inflammation markers and altered adipokines concentrations^11^. Data concerning C-reactive protein (CRP), a classic inflammation marker, are controversial. Several studies showed higher CRP concentrations in PCOS women than in controls^12,13^, while other authors report no significant differences when comparing both groups of women^14,15^.

Among metabolic alterations that can be found in PCOS women, vitamin D deficiency has been described, in relationship with the pathogenesis of insulin resistance and metabolic risk factors in this disease^16,17^. Moreover, some authors found that vitamin D supplementation in PCOS deficient women improves their metabolic profile, although data are controversial^18,19^. Beyond its classical role in bone metabolism, vitamin D actions in several tissues are described and it has been related to different diseases. Vitamin D receptors can be found in adipose tissue, muscle, pancreatic β cells^20^, as well as in ovary and male genital tract^21^. In ovary, these receptors have been found in granulose cells’ nucleus and cytoplasm, suggesting a role of vitamin D in female reproduction^22^.

On the other hand, it is described that a hyperfunction of the hypothalamic-pituitary-adrenal axis could be programmed from early stages of life, as a consequence of adverse conditions for endometrial environment during pregnancy, which are reflected with alterations like low birth weight. This condition is related to endocrinological *sequelae*, as premature adrenarchia and PCOS^23^. This adrenal hyperfunction programming, that could be crucial for fetus survival in hostile intrauterine conditions, could lead to obesity and hyperandrogenism in childhood and probably in adult life^24^. However, data about adrenal axis status in relationship to the association of glucocorticoids and low birth weight in hyperandrogenic diseases like PCOS are scarce in the literature.

The aim of this study was to establish the relationships between metabolic and inflammatory markers with vitamin D levels and adrenal parameters in PCOS adult women.

## Methods

### Study subjects

We studied 73 adult women (18–44 years), with diagnosis of PCOS according to the Rotterdam criteria, who were selected at the Gynecological and the Endocrinology Divisions, Hospital de Clínicas, University of Buenos Aires and Endocrinology Service, Hospital Militar Central, Buenos Aires, Argentina. Pregnant women were not included, as well as those receiving oral contraceptives, hormonal replacement therapy, corticoids or any other drug modifying lipid metabolism in the previous 3 months. Women with diabetes, renal, hepatic or thyroid disorders, hormone dependent tumors, vaginal bleeding of unknown etiology and cardiovascular disease, were also excluded. In no case did alcohol consumption surpass 10 g/day and none of them was smoker. We also recruited a control group of 33 healthy women (22–45 years), with regular menstrual cycles, with no hyperandrogenemia or hirsutism and without pharmacological treatment. All women gave their informed consent and the study protocol was approved by the Ethics Committee of the Hospital de Clínicas, Universidad de Buenos Aires. The study was conducted in accordance with the ethical standards of the institutional research committee and with the 1964 Helsinki declaration and its later amendments or comparable ethical standards.

### Samples

Blood samples were collected by vein puncture in the follicular phase of the menstrual cycle (days 3–5), after 12 hours fasting and between 8 and 9 a.m., either in PCOS patients or in controls. In those women in amenorrhea, blood was drawn at random. For hormonal determinations and high sensitive CRP (hs-CRP) serum was stored at −70 °C; glucose and triglycerides were measured the same day of extraction.

### Anthropometric parameters

The weight and height of each patient were measured and body mass index (BMI) was calculated as weight (kg)/height (m)^2^ to evaluate the obesity degree. Waist circumference (WC) was measured as an indicator of abdominal obesity, at the midway level between the lateral lower rib margin and the superior anterior iliac crest, in a standing position.

### Biochemical parameters

All the methods described were carried out in accordance with the relevant guidelines and regulations. Triglycerides (TG) and glucose were measured by enzymatic colorimetric methods in a Cobas 6000 autoanalyzer (Roche Diagnostic Corporation, Indianapolis, USA). These measurements were conducted under good quality control (coefficient of variation (CV) routinely <2% in every case). Total testosterone (TT) and androstenedione (A4) were determined by radioimmunoassay (DIA Source ImmunoAssays SA), CVs intra and interasssay lower than 6.2% and 9% respectively; analytical sensitivity 0,05 ng/ml and 0.03 ng/ml, respectively. Bioavailable testosterone (bioT) was calculated from TT and SHBG through Vermeulen’s equation^25^. SHBG and serum cortisol were measured through a chemoluminescent method (Immulite 2000 autoanalyzer, Siemens Healthcare Diagnostics Products Lld. United Kingdom), CV intra and inter-assay lower than 13.5% and 9.4%, respectively. Insulin was determined by an immunoradiometric assay (DIA Source ImmunoAssays SA), analytical sensitivity 1 uUI/ml, CV intra and interassay lower than 6.5%. Insulin resistance was determined through the HOMA (Homeostasis Model Assessment of Insulin Sensitivity) index^26^. hs-CRP was determined by an inmunoturbidimetric method in a Cobas 6000 autoanalyzer (Roche Diagnostic Corporation, Indianapolis, USA), analytical sensitivity 0.3 mg/L, CV% lower than 8.4%. The LAP index was calculated using the formula: [waist (cm) − 58] × triglyceride (mmol/l), as previously reported^9^. Vitamin D status was determined by assessing circulating levels of 25-hydroxyvitamin D (25-OH-D) through a direct chemoluminescent method (Advia Centaur® XP), total CV% lower than 11.9 and limit of quantitation 4.2 ng/ml.

### Statistical analysis

In order to evaluate differences between groups parametric and non-parametric methods were used, according to data distribution (Student t test and Mann-Whitney test respectively). Correlations between variables were calculated using Pearson or Spearman test. Binary logistic regression analysis was performed to test the chance that one event (hyperandrogenism) occurs in association with other factors, after doing the corresponding corrections and also to test which variables were associated to PCOS development. A *p* value < 0.05 was considered statistically significant. Statistical analysis was performed using SPSS 22 software and GraphPad Prism 3.0.

## Results

Mean women’s age in control group was significantly higher than in patients; however both groups showed a similar range in this parameter (18–44 years in patients *vs* 22–45 years in controls). When comparing with the control group, PCOS women showed higher BMI, WC, TT, bioT, A4, insulin, HOMA, hs-CRP and LAP index. Regarding this last parameter, median in the study group was higher than 34.5, cut-off value established by Wiltgen *et al*.^10^ to identify insulin resistant patients with an adequate sensitivity and specificity. On the other hand, SHBG and 25-OH-D levels were lower in patients than in controls and serum cortisol showed no significant differences between groups (Table 1). Considering that many of the PCOS patients were overweight or obese, we performed binary logistic regression analysis to evaluate if the differences found in the parameters studied were attributable to PCOS itself or to differences in body weight and fat distribution. Table 2 shows that TT, bioT, A4, insulin and HOMA continued being significantly different between groups after adjusting by BMI and WC but this correction showed that differences in SHBG, hs-CRP, LAP and 25-OH-D were due to body weight and fat distribution.

**Table 1:** Antropometric parameters, hormonal and metabolic profile and hs-CRP levels in the population studied.

	PCOS (n = 73)	Control (n = 33)	*p*
Age (years)	26,9 ± 5,5	30,9 ± 5,1	0,001
BMI (kg/m2)	31,0 (18,6–46,9)	22,4 (18,1–37,4)	<0,0001
WC (cm)	102 (69–133)	81 (52–110)	<0,0001
TT (nmol/L)	2,88 ± 1,18	1,70 ± 0,42	<0,0001
bioT (nmol/L)	1,14 (0,35–2,84)	0,49 (0,28–0,87)	<0,0001
A4 (nmol/L)	11,5 ± 4,5	8,0 ± 4,5	<0,0001
SHBG (nmol/L)	26,6 (8,2–105)	61 (19,4–121)	<0,0001
Insulin (µUI/ml)	12,7 (4,2–65,2)	6,8 (2,2–14,4)	<0,0001
HOMA	3,46 (0,89–17,86)	1,51 (0,47–3,48)	<0,0001
hs-CRP (mg/L)	2,38 (0,36–14,53)	1.11 (0,27–9,30)	0,005
LAP (cm.mmol/L)	53,5 (5,0–203,8)	17,4 (6,8–93,4)	<0.0001
25-OH-D (nmol/L)	28,8 (25–64)	42,8 (62,5–97)	0,020
Cortisol (nmol/L)	342,1 ± 129,7	369,7 ± 102,1	ns

Results are expressed as median (range) or mean ± SD according to distribution. BMI: body mass index, WC: waist circumference, TT: total testosterone, bioT: bioavailable testosterone, A4: androstenedione, hs-CRP: high sensitive C reactive protein, LAP: lipid accumulation product, 25-OH-D: 25-hydroxyvitamin D.

**Table 2:** Binary logistic regression analysis for the different parameters studied after adjusting by BMI and WC.

Parameter	*p*	RR
TT	<0,0001	34897,945
bioT	0,001	4,187 × 10^8^
A4	0,001	4,224
SHBG	0,089	0,982
Insulin	0,010	1,332
HOMA	0,019	2,982
hs-CRP	0,639	1,050
LAP	0,146	1,131
25-OH-D	0,534	0,942

BMI: body mass index, WC: waist circumference, TT: total testosterone, bioT: bioavailable testosterone, A4: androstenedione, hs-CRP: high sensitive C reactive protein, LAP: lipid accumulation product, 25-OH-D: 25-hydroxyvitamin D.

25-OH-D was negatively associated with BMI, WC, TT, bioT, hs-CRP and LAP index, while hs-CRP showed a positive correlation with BMI, WC, bioT, insulin, HOMA and LAP and negatively correlated with SHBG. Additionally, LAP index positively correlated with BMI, WC, TT, bioT, insulin and HOMA and negatively correlated with SHBG (Table 3).

**Table 3:** Correlations between 25-OH-D, hs-CRP and LAP index with different parameters.

	25-OH-D	hs-CRP	LAP
BMI	r = −0,416, p = 0,002	r = 0.527, p < 0.0001	r = 0.813, p < 0.0001
WC	r = −0,57, p < 0,0001	r = 0.525, p < 0.0001	r = 0.894, p < 0.0001
TT	r = −0,299, p = 0,030	ns	r = 0.364, p = 0.001
bioT	r = −0,349, p = 0,016	r = 0.307, p = 0.005	r = 0.595, p < 0.0001
SHBG	ns	r = −0.286, p = 0.009	r = −0.667, p < 0.0001
Insulin	ns	r = 0.535, p < 0.0001	r = 0.767, p < 0.0001
HOMA	ns	r = 0.537, p < 0.0001	r = 0.752, p < 0.0001
hs-CRP	r = −0,292, p = 0,034	—	r = 0.546, p < 0.0001
LAP	r = −0,551, p < 0,0001	r = 0.546, p < 0.0001	—

25-OH-D: 25-hydroxyvitamin D, hs-CRP: high sensitive C reactive protein, LAP: lipid accumulation product, BMI: body mass index, WC: waist circumference, TT: total testosterone, bioT: bioavailable testosterone, ns: non-significant.

In turn, cortisol was positively associated with A4 (r = 0.447, p < 0.0001). A binary logistic regression analysis showed that cortisol and 25-OH-D were associated to PCOS development (p = 0.015; RR = 0.617; 95% CI [0.417–0.912] and p = 0.015; RR = 0.808; 95% CI [0.680–0.960] respectively), even when A4 was introduced in the model.

## Discussion

In this study we analyzed the possible associations between metabolic alterations, androgenic and adrenal parameters and inflammation markers in PCOS adult women.

As well as other authors, we found lower vitamin D levels in PCOS patients than in control group and vitamin D inversely correlated with TT, bioT and with LAP index, a secondary marker of insulin resistance. As well, the regression analysis showed that vitamin D was associated to PCOS development. However, taking into account that difference in vitamin D between groups was attributable to BMI and WC, more studies are needed to understand the role that vitamin D should play in PCOS.

Several studies demonstrated inverse associations between vitamin D and inflammation markers^27,28^. Considering that vitamin D modulates the immune system, low levels can induce an inflammatory response^29^. Nowadays PCOS is considered a low grade chronic inflammation state, with high serum levels of tumor necrosis factor - α (TNF-α), interleukin-6 and CRP, that could be the link between PCOS and its long-term complications like type 2 diabetes and cardiovascular disease^11^. The inverse correlation found between vitamin D and hs-CRP levels in our group of patients is in accordance with these observations.

Regarding CRP, some authors reported high levels in PCOS patients^12,30^, also related to insulin resistance^30,31^, agreeing with our results. Moreover, in this study, hs-CRP levels showed an inverse correlation with SHBG and a positive association with bioT. However, after correcting by BMI and WC, the difference in hs-CRP levels between PCOS and control women was not significant, showing that overweight and abdominal obesity could be the major determinants of this inflammation marker in PCOS patients. These results are in accordance with those of Ganie *et al*.^32^, who suggested that in Indian adolescent women, hs-CRP levels may not *per se* be associated with PCOS, but could be related to fat mass. According to the results of Lee *et al*.^33^, we also found that hs-CRP positively correlated with HOMA and LAP.

We found that LAP index was significantly higher in PCOS than in control women and it strongly correlated with insulin and HOMA index. The significant increased LAP index value in PCOS patients is in accordance with literature concerning this index as a good indicator of insulin resistance states^34,35^. Moreover, Nascimento *et al*.^36^ considered that LAP index is an adequate indicator of cardiovascular disease risk in women with PCOS. However, after the regression analysis adjusting results by BMI and WC, difference in LAP index between groups was not significant. This was expected given that WC is included in LAP equation. Reinforcing the fact that hyperandrogenism is related to insulin resistance^37,38^, we also found that LAP was positively associated with TT and bioT.

As expected, differences in SHBG levels between PCOS women and controls were due to body weight and abdominal obesity.

Analyzing if there was any association between adrenal parameters and androgens, we found that cortisol showed a positive and strong correlation with A4, an androgen that is frequently elevated in PCOS patients. It has been described that fetal or neonatal exposure to excessive glucocorticoid levels, as well as exposure in early development, can lead to functional alterations later in life or the onset of certain chronic diseases^39^, in accordance with the critical role of adrenal axis in cardiovascular, reproductive, metabolic and neurological systems. Maternal, fetal and neonatal endogen glucocorticoid levels could be high because of maternal malnutrition, depression, glucocorticoid treatment, as well as fetal or neonatal stress. This prenatal exposure to elevated glucocorticoid concentrations could program a hyperactivation of adrenal axis and long-term development of chronic illnesses^40^. On the other hand, adverse conditions to endometrial environment during pregnancy, which can lead to low birth weight, could modulate adrenal glands function, leading to obesity and hyperandrogenism in childhood and probably in adult life^24^. Moreover, endocrinological *sequelae* of intrauterine growth retardation include short stature in children and adults, premature adrenarchia and PCOS^41^.

An additional interesting data is that although in this study we did not find any differences in plasmatic cortisol levels between patients and controls, in a subgroup of 24 PCOS patients we found that the median of hair cortisol was significantly higher than the cut-off value of a normal population (data not shown). Recently, hair cortisol measurement showed that it provides a retrospective index of integrated cortisol secretion over periods of several months^42^ and it has been proposed as the best biomarker to evaluate adrenal axis as well as a potential biomarker of chronic stress^43^.

In conclusion, the fact that vitamin D levels were lower in PCOS patients than in controls, along with its association with PCOS development and its correlations with androgenic and metabolic parameters, suggests its evaluation in PCOS patients. Correlations found between LAP index and insulin resistance parameters, as well as with hs-CRP, confirm that it is a good indicator of metabolic complications and it could be clinically useful in PCOS. On the other hand, although cortisol levels were not different between PCOS patients and the control group, the fact that it was associated with PCOS development merits a new study in order to analyze the perinatal history of the patients and its possible relationship with a hyperactivation of the adrenal axis in adult life.

## Data Availability

The datasets generated during and/or analyzed during the current study are available from the corresponding author on reasonable request.
